# Common factors influencing childhood undernutrition and their comparison between Sylhet, the most vulnerable region, and other parts of Bangladesh: Evidence from BDHS 2007–18 rounds

**DOI:** 10.3389/fnut.2022.999520

**Published:** 2023-01-09

**Authors:** Kazi Istiaque Sanin, Mansura Khanam, Razia Sultana Rita, Md. Ahshanul Haque, Tahmeed Ahmed

**Affiliations:** Nutrition and Clinical Services Division, International Centre for Diarrhoeal Disease Research, Bangladesh, Dhaka, Bangladesh

**Keywords:** undernutrition, stunting, wasting, underweight, Sylhet region, Bangladesh, trend

## Abstract

**Introduction:**

Undernourishment is disproportionately spread within Bangladesh, making some regions like Sylhet more vulnerable than the rest of the country. We aimed to assess the trend of diverse associated factors related to childhood stunting, wasting, and being underweight. Furthermore, we have compared the estimated factors between Sylhet, the most vulnerable region, and other parts of Bangladesh.

**Methods:**

We performed a secondary data analysis where data were derived from the nationally representative cross-sectional surveys: Bangladesh demographic and health survey (BDHS) 2007, 2011, 2014, and 2017–18 rounds. The outcome variables were childhood undernutrition, including stunting, wasting, and being underweight. Descriptive statistics such as mean, standard deviation, frequency, and proportion were used to summarize the data. All variables were summarized by BDHS survey time points. We used multiple logistic regression models to measure the associated factors with childhood stunting, wasting, and being underweight.

**Results:**

The percentage of children under the age of 5 years who were stunted declined from 40% in 2007 to 31% in 2018. Similar trends are observed in the decrease in the percentage of underweight children, dropping from 39% in 2007 to 22% in 2018. Wasting dropped to 8% in 2018 after years of critically high levels (17%). According to the results of the regression analyses, urban residence, child’s age and gender, morbidity, maternal BMI, maternal and paternal education, decision-making ability, use of contraceptives, the occurrence of domestic violence, antenatal care, c-section, and birth interval, as well as geographic region, were all linked to childhood malnutrition.

**Conclusion:**

The Sylhet division falls short in several critical associated indicators, including parental education, maternal BMI, obtaining at least four ANC, women empowerment, and usage of contraceptives. Policymakers must concentrate on region-specific planning and proper intervention to achieve a more uniform improvement across the country.

## Background

Undernutrition includes wasting (low weight-for-height), stunting (low height-for-age), and underweight (low weight-for-age). Low weight-for-height is known as wasting, indicating acute and severe weight loss. Low height-for-age or stunting is a consequence of chronic undernutrition. Underweight or low-weight-for-age children are a combination of the above two outcomes and can be stunted, wasted, or both (1, 2). Undernourished children face greater vulnerability to disease and subsequent death as nearly 45% of deaths among children under 5 years of age could be directly related to this (3). A significant portion of these children come from low- and middle-income countries. However, the prevalence of undernourished children greatly varies within the same country. This is well evident that undernutrition is an adverse outcome modulated by multidimensional components (4). These encompass direct factors such as poor dietary habits and illness (5); lack of food security and inadequate water, sanitation and hygiene commodities (6); insufficient access to health services (7) and overarching social factors like poverty with economic and demographic disadvantages (8). All these elements vary between and within countries, which requires context-specific research to develop interventions supported by evidence (9).

Bangladesh has achieved impressive social and economic development growth over the past three decades. Yet, a hefty proportion of its population (43%) lives under the poverty line of 1.25 dollars per day (10). Such a vast population unable to afford nutritious food or access improved healthcare facilities, makes the goal of reducing the country’s prevalence of undernourished children extremely challenging. Furthermore, undernourishment is disproportionately spread within the country, making some regions even more vulnerable than others (11, 12). Although the poverty rate is lower in the eastern part of Bangladesh, surprisingly, the undernutrition rate is much more prevalent in this region compared to the north-western part (12, 13). The Sylhet region is considered ecologically susceptible due to being a remote area, wetland ecosystems, and social dogmatism (7, 13). Such unique regional factors may create non-income obstacles, hindering the nutritional status of children (12). However, the change in these factors in Bangladesh over time has yet to be explored. Through our analysis, we aimed to assess the trend of childhood stunting, wasting, and underweight and explore the factors influencing these metrics. The second objective was to compare the estimated factors between Sylhet, the most vulnerable region, and other regions of Bangladesh.

## Materials and methods

### Data sources

For this paper, we used secondary data derived from the nationally representative cross-sectional surveys of the BDHS 2007 (14), 2011 (15), 2014 (16), and 2018 (17), undertaken by the authority of the Ministry of Health and Family Welfare’s National Institute for Population Research and Training (NIPORT). The BDHS follows a similar study design that has been described in the published reports. Briefly, the BDHS sample was stratified and selected in two stages. Administratively, Bangladesh was divided into several divisions, and each division was further stratified into urban and rural areas. The whole list of enumeration areas (EAs) spanning the entire nation, created by the Bangladesh Bureau of Statistics for the People’s Republic of Bangladesh population census, served as the sample frame for the BDHS.

To draw the sample, required EAs as a cluster were selected with a probability proportional to the EA in the first stage. Household listing was done based on the inclusion and exclusion criteria and prepared the sampling frame. In the second stage of selection, a fixed number of required households per cluster were selected using a systematic sampling procedure from the newly created sampling frame. We used Children’s Record (KR) data for this analysis. Data for a total of 12,860 youngest children were used from four consecutive BDHS (2007–2011); of those 4,926 were from 2007, 7,325 samples from 2011, 6,855 samples from 2014, and the rest of the 7,562 were extracted from the 2017-18 BDHS.

### Variable under study

The outcome variables of this paper focus on childhood undernutrition including stunting, wasting, and being underweight. These variables were derived from child’s age and sex-specific composite indicators such as length/height-for-age z score (LAZ/HAZ), weight-for-length/height z score (WLZ/WHZ) and weight-for-age z score where the z score was defined as “(observe anthropometry value – average value of reference population)/standard deviation of reference population.” Children were defined as stunted if LAZ/HAZ < −2, wasted if WLZ/WHZ < −2 and underweight if WAZ < −2 [2]. In the database, cases were treated as missing if LAZ/HAZ > 6 or LAZ/HAZ < −6, WLZ/WHZ > 5 or WLZ/WHZ < −5, and WAZ > 5 or WAZ < −6.

Based on the literature review as well as the bi-variate relationship with childhood nutritional status, several independent variables were selected such as geographical area, place of residence, wealth index, improved toilet, source of drinking water, religion, maternal BMI < 18.5, maternal education, empowerment, attitude toward domestic violence, receiving at least four ANC from a medically trained provider, delivery type, use of a contraceptive method, paternal education, birth interval, current age – respondent, partners age, child’s age in months, child’s sex, and having fever in last 2 weeks. The mother’s empowerment was defined as the ability to make decisions about her own health care, major household purchases, and visits to family or relatives.

### Statistical analyses

We performed analyses using Stata version 13.0 (StataCorp, College Station, TX, USA). Firstly, to visualize the outcome indicators, statistical plot like bar diagram was used. Several descriptive statistics such as mean, standard deviation, frequency, and proportion were used to summarize the data. All variables were summarized by BDHS survey time points. Due to binary outcomes, simple logistic regression was used to assess the bi-variate association between outcome variables and all independent variables. We used multiple logistic regression models to assess the associated factors with childhood stunting, wasting, and being underweight. The independent variables were included in the model based on the literature review as well as the bi-variate association. “svyset” option was used to allow for adjustments for the cluster sampling design, weights and the calculation of standard errors. Again, logistic regression was used to assess the status of wealth index, birth interval, cesarean delivery, ANC visit, attitude toward domestic violence, contraceptive methods, empowerment, paternal and maternal education, maternal underweight, and child’s morbidity in the Barisal, Chittagong, Dhaka, Khulna, and Rajshahi regions compared with the Sylhet region. We selected the Sylhet region as the reference as several health indicators are performing poorly here. The odds ratios with 95% CIs were calculated as inferential statistics and *P* < 0.05 were considered as a significance level.

## Results

**FIGURE 1:**
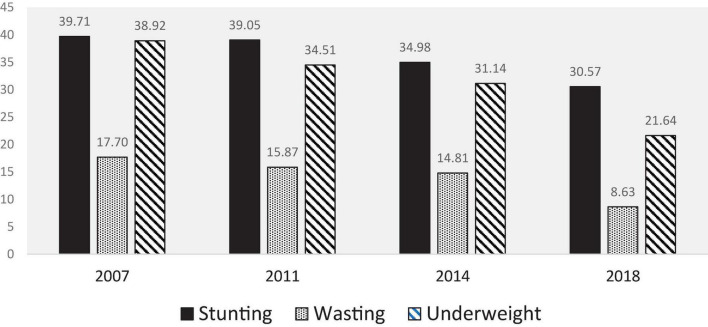
Proportion of childhood stunting, wasting, and underweight over the period.

Over 60% of the data was collected from rural areas, and the rest of the data was collected from urban areas over time. The background characteristics of the surveyed children from 0 to 59 months old in 2007, 2011, 2014, and 2018 BDHS are shown in Table 1. Our findings show the nutritional status of children has improved steadily over the past decade (Figure 1). The percentage of children under age of 5 years who were stunted declined from 40% in 2007 to 31% in 2018. The decline in the percentage of children who are underweight followed a similar pattern, falling from 39% in 2007 to 22% in 2018. After years at critically high levels (17%), wasting decreased to 8% in 2018.

**TABLE 1:** General characteristics of households by survey time points.

Indicators, *n* (%)	2007	2011	2014	2018
Geographical area				
Barisal	658 (13.4)	856 (11.7)	814 (11.9)	790 (10.4)
Chittagong	980 (19.9)	1393 (19)	1284 (18.7)	1224 (16.2)
Dhaka^1^	1051 (21.3)	1229 (16.8)	1222 (17.8)	2022 (26.7)
Khulna	623 (12.6)	877 (12)	778 (11.3)	828 (10.9)
Rajshahi^2^	829 (16.8)	1911 (26.1)	1732 (25.3)	1698 (22.5)
Sylhet	785 (15.9)	1059 (14.5)	1025 (15)	1000 (13.2)
Place of residence				
Urban	1748 (35.5)	2328 (31.8)	2215 (32.3)	2701 (35.7)
Rural	3178 (64.5)	4997 (68.2)	4640 (67.7)	4861 (64.3)
Wealth index				
Poorest	937 (19)	1526 (20.8)	1435 (20.9)	1610 (21.3)
Poorer	988 (20.1)	1400 (19.1)	1295 (18.9)	1467 (19.4)
Middle	910 (18.5)	1408 (19.2)	1332 (19.4)	1369 (18.1)
Richer	943 (19.1)	1473 (20.1)	1412 (20.6)	1534 (20.3)
Richest	1148 (23.3)	1518 (20.7)	1381 (20.1)	1582 (20.9)
Improved toilet (yes)	1906 (40.3)	3506 (49.5)	4286 (63.9)	4186 (58.7)
Source of drinking water (improved)	4280 (87.2)	6456 (88.1)	6078 (88.9)	6486 (86.4)
Religion				
Islam	4473 (90.8)	6600 (90.1)	6279 (91.6)	6892 (91.1)
Hinduism	419 (8.5)	699 (9.5)	523 (7.6)	624 (8.3)
Buddhism	17 (0.3)	14 (0.2)	39 (0.6)	32 (0.4)
Christianity	12 (0.2)	12 (0.2)	13 (0.2)	14 (0.2)
Other	4 (0.1)	0 (0)	1 (0)	0 (0)
Maternal BMI < 18.5	1514 (31.1)	1906 (26.6)	1513 (22.2)	1070 (14.4)
Maternal education				
No education	1268 (25.7)	1332 (18.2)	1040 (15.2)	531 (7)
Primary	1507 (30.6)	2193 (29.9)	1871 (27.3)	2132 (28.2)
Secondary	1742 (35.4)	3174 (43.3)	3189 (46.5)	3598 (47.6)
Higher	406 (8.2)	626 (8.5)	755 (11)	1301 (17.2)
Woman’s own health care	2703 (54.9)	4464 (62)	4183 (61.9)	5542 (74.7)
Making major household purchases	2557 (51.9)	4066 (56.5)	3786 (56.1)	5133 (69.2)
Visits to her family or relatives	2672 (54.2)	4286 (59.6)	3914 (58)	5327 (71.8)
None of the three decisions	1410 (28.6)	1794 (24.9)	1708 (25.3)	1024 (13.8)
Less occurrence domestic violence	3329 (67.6)	4941 (67.5)	4876 (71.1)	6184 (81.8)
At least 4 ANC from medically trained provider	997 (20.2)	1621 (22.1)	1182 (17.2)	2246 (29.7)
Delivery type				
Cesarean section	478 (9.7)	1168 (16)	1088 (24.2)	1671 (33.4)
Non-cesarean	4446 (90.3)	6146 (84)	3405 (75.8)	3336 (66.6)
Use contraceptive method	2853 (57.9)	4882 (66.6)	4702 (68.6)	5172 (68.4)
Paternal education				
No education	1585 (32.2)	1959 (26.7)	1692 (24.7)	1096 (14.8)
Primary	1380 (28)	2126 (29)	2044 (29.8)	2469 (33.3)
Secondary	1319 (26.8)	2194 (30)	2098 (30.6)	2413 (32.5)
Higher	636 (12.9)	1040 (14.2)	1019 (14.9)	1422 (19.2)
Birth interval				
No previous birth	1543 (31.4)	2476 (33.9)	2575 (37.6)	2758 (36.6)
<24 months	478(9.7)	552 (7.6)	462 (6.8)	509 (6.8)
≥24 months	2895 (58.9)	4276 (58.5)	3803 (55.6)	4272 (56.7)
Current age – respondent^†^	25.9 ± 6.34	25.7 ± 6.06	25.6 ± 5.96	25.9 ± 5.8
Partners age^†^	35.2 ± 8.68	34.6 ± 8.88	34 ± 7.88	33.8 ± 7.26
Child’s age in months^†^	26.5 ± 16.44	27.7 ± 17.21	27.5 ± 16.72	27.3 ± 17.21
Child’s sex				
Male	2515 (51.1)	3790 (51.7)	3576 (52.2)	3973 (52.5)
Female	2411 (48.9)	3535 (48.3)	3279 (47.8)	3589 (47.5)
Child had fever in last 2 weeks	1847 (38.8)	2743 (38.6)	2518 (37.8)	2519 (34.1)

^1^Mymensingh was merged with Dhaka, ^2^Rangpur was merged with Rajshahi, and ^†^mean ± SD.

Table 2 presents the factors associated with childhood stunting, wasting and being underweight. Childhood stunting was associated with urban residence [aOR: 1.12 (95% CI: 1.00, 1.25); *p*-value = 0.047], having a fever in the last 2 weeks [aOR: 1.17 (95% CI: 1.08, 1.26); *p*-value < 0.001], maternal BMI < 18.5 [aOR: 1.32 (95% CI: 1.22, 1.44); *p*-value < 0.001], maternal education below secondary [aOR: 1.16 (95% CI: 1.05, 1.27); *p*-value = 0.002], paternal education below secondary [aOR: 1.34 (95% CI: 1.23, 1.46); *p*-value < 0.001], not having decision-making power [aOR: 1.11 (95% CI: 1.02, 1.22); *p*-value = 0.022], using contraceptive [aOR: 1.21 (95% CI: 1.12, 1.31); *p*-value < 0.001], the occurrence of domestic violence [aOR: 1.09 (95% CI: 1.01, 1.18); *p*-value = 0.028], not receiving at least four ANC [aOR: 1.13 (95% CI: 1.02, 1.26); *p*-value = 0.017], non-cesarean delivery [aOR: 1.31 (95% CI: 1.18, 1.47); *p*-value < 0.001], and birth interval < 24 months [aOR: 1.22 (95% CI: 1.03, 1.44); *p*-value = 0.018].

**TABLE 2:** Factors associated with childhood stunting, wasting and underweight.

	Stunting		Wasting		Underweight	
	**aOR (95 CI)**	***P*-value**	**aOR (95 CI)**	***P*-value**	**aOR (95 CI)**	***P*-value**
Geographical region						
Sylhet	Reference		Reference		Reference	
Barisal	0.85 (0.73, 0.98)	0.025	1.00 (0.81, 1.23)	0.981	0.86 (0.73, 1.02)	0.080
Chittagong	0.89 (0.78, 1.02)	0.090	1.11 (0.93, 1.31)	0.249	0.96 (0.84, 1.10)	0.568
Dhaka	0.84 (0.74, 0.96)	0.013	0.95 (0.79, 1.14)	0.569	0.80 (0.69, 0.93)	0.004
Khulna	0.68 (0.59, 0.78)	0.000	1.02 (0.85, 1.22)	0.824	0.72 (0.61, 0.84)	0.000
Rajshahi	0.72 (0.63, 0.82)	0.000	0.99 (0.83, 1.18)	0.907	0.82 (0.70, 0.95)	0.007
Place of residence						
Rural	Reference		Reference		Reference	
Urban	1.12 (1.00, 1.25)	0.047	0.99 (0.87, 1.11)	0.814	1.02 (0.92, 1.13)	0.676
Child’s sex						
Female	Reference		Reference		Reference	
Male	1.07 (0.99, 1.15)	0.097	1.16 (1.05, 1.29)	0.005	0.96 (0.89, 1.04)	0.310
Child’s age in months	1.02 (1.02, 1.03)	0.000	1.00 (1.00, 1.00)	0.514	1.02 (1.02, 1.03)	0.000
Having fever						
No	Reference		Reference		Reference	
Yes	1.17 (1.08, 1.26)	0.000	1.30 (1.17, 1.45)	0.000	1.38 (1.28, 1.48)	0.000
Maternal BMI < 18.5						
BMI ≥ 18.5	Reference		Reference		Reference	
BMI < 18.5	1.32 (1.22, 1.44)	0.000	1.59 (1.42, 1.77)	0.000	1.79 (1.64, 1.94)	0.000
Maternal education						
At least secondary	Reference		Reference		Reference	
Below secondary	1.16 (1.05, 1.27)	0.002	1.30 (1.16, 1.45)	0.000	1.29 (1.18, 1.41)	0.000
Religion						
Muslim	Reference		Reference		Reference	
Others	0.92 (0.80, 1.05)	0.206	0.95 (0.82, 1.11)	0.539	1.03 (0.89, 1.18)	0.717
Paternal education						
At least secondary	Reference		Reference		Reference	
Below secondary	1.34 (1.23, 1.46)	0.000	0.94 (0.84, 1.07)	0.355	1.22 (1.09, 1.35)	0.000
Decision making power						
At least one						
None of three*	1.11 (1.02, 1.22)	0.022	0.96 (0.85, 1.08)	0.473	1.05 (0.96, 1.15)	0.310
Contraceptive						
No	Reference		Reference		Reference	
Yes	1.21 (1.12, 1.31)	0.000	0.95 (0.86, 1.05)	0.292	1.14 (1.04, 1.24)	0.005
Domestic violence						
No	Reference		Reference		Reference	
Yes	1.09 (1.01, 1.18)	0.028	1.02 (0.92, 1.13)	0.759	1.01 (0.93, 1.09)	0.841
At least 4 ANC from medically trained provider						
Yes	Reference		Reference		Reference	
No	1.13 (1.02, 1.26)	0.017	1.10 (0.97, 1.26)	0.149	1.22 (1.09, 1.37)	0.000
Mode of delivery						
Cesarean	Reference		Reference		Reference	
Non-cesarean	1.31 (1.18, 1.47)	0.000	1.08 (0.91, 1.28)	0.391	1.22 (1.08, 1.39)	0.002
Birth interval						
No previous birth	Reference		Reference		Reference	
<24 months	1.22 (1.03, 1.44)	0.018	1.02 (0.85, 1.23)	0.801	1.26 (1.05, 1.51)	0.012
≥24 months	0.95 (0.88, 1.03)	0.238	0.98 (0.88, 1.10)	0.773	0.97 (0.88, 1.07)	0.555
Wealth index						
Richest	Reference		Reference		Reference	
Poorest	2.01 (1.79, 2.45)	0.000	1.19 (0.97, 1.46)	0.088	2.00 (1.69, 2.37)	0.000
Poorer	1.85 (1.60, 2.14)	0.000	1.05 (0.87, 1.27)	0.631	1.64 (1.38, 1.96)	0.000
Middle	1.61 (1.40, 1.85)	0.000	1.03 (0.86, 1.24)	0.720	1.43 (1.21, 1.68)	0.000
Richer	1.40 (1.22, 1.60)	0.000	1.07 (0.89, 1.28)	0.457	1.25 (1.08, 1.44)	0.003
Round						
2007	Reference		Reference		Reference	
2011	0.94 (0.85, 1.05)	0.287	0.92 (0.81, 1.04)	0.197	0.82 (0.74, 0.90)	0.000
2014	0.89 (0.79, 1.01)	0.075	0.95 (0.82, 1.10)	0.460	0.85 (0.75, 0.97)	0.013
2018	0.95 (0.84, 1.08)	0.441	0.50 (0.42, 0.59)	0.000	0.56 (0.49, 0.63)	0.000

*Woman’s own health care, making major household purchases, and visits to her family or relatives. Adjusted odds ratios (aOR) were estimated using multiple logistic regression analysis. The outcome variables were stunting, wasting, underweight, and independent variables were the indicators given in the first column.

Childhood wasting was associated with the male sex [aOR: 1.16 (95% CI: 1.05, 1.29); *p*-value = 0.005], having a fever in the last 2 weeks [aOR: 1.30 (95% CI: 1.17, 1.45); *p*-value < 0.001], maternal BMI < 18.5 [aOR: 1.59 (95% CI: 1.42, 1.77); *p*-value < 0.001], and maternal education below secondary [aOR: 1.30 (95% CI: 1.16, 1.45); *p*-value < 0.001].

Childhood underweight was associated with having fever in the last 2 weeks [aOR: 1.38 (95% CI: 1.28, 1.48); *p*-value < 0.001], maternal BMI < 18.5 [aOR: 1.79 (95% CI: 1.64, 1.94); *p*-value < 0.001], maternal education below secondary [aOR: 1.29 (95% CI: 1.18, 1.41); *p*-value < 0.001], paternal education below secondary [aOR: 1.22 (95% CI: 1.09, 1.35); *p*-value < 0.001], using contraceptive [aOR: 1.14 (95% CI: 1.04, 1.24); *p*-value = 0.005], not receiving at least four ANC [aOR: 1.22 (95% CI: 1.09, 1.37); *p*-value < 0.001], non-cesarean delivery [aOR: 1.22 (95% CI: 1.08, 1.39); *p*-value = 0.002], and birth interval < 24 months [aOR: 1.26 (95% CI: 1.05, 1.51); *p*-value = 0.012]. Geographical region and child’s age were associated with both stunting and underweight. On the other hand, low socio-economic status had high prevalence of childhood stunting and being underweight.

We have compared the common predictors of undernutrition in Table 3 across the geographical regions. We found that status of cesarean delivery, receiving at least 4 ANC from a medically trained provider, use of contraceptive methods, empowerment, paternal education, maternal education, maternal BMI > 18.5, and not reporting fever in the last 2 weeks were better in those regions compared with the Sylhet region.

**TABLE 3:** Comparison of the predictors of malnutrition among the geographical regions.

Indicators	^†^Adjusted OR (95% CI) for geographic areas compared to Sylhet				
	**Barisal**	**Chittagong**	**Dhaka**	**Khulna**	**Rajshahi**
Birth interval^1^					
<24 months	0.37 (0.29, 0.46)*	0.52 (0.42, 0.63)*	0.40 (0.33, 0.48)*	0.24 (0.19, 0.30)*	0.31 (0.25, 0.37)*
≥24 months	0.72 (0.63, 0.83)*	0.83 (0.73, 0.95)*	0.73 (0.64, 0.83)*	0.62 (0.54, 0.70)*	0.72 (0.63, 0.82)*
Cesarean delivery	1.08 (0.85, 1.37)	1.19 (0.95, 1.47)	1.84 (1.51, 2.25)*	2.47 (2.03, 3.02)*	1.43 (1.18, 1.74)*
At least 4 ANC from medically trained provider	1.17 (0.95, 1.45)	1.16 (0.92, 1.47)	1.30 (1.06, 1.61)*	1.88 (1.53, 2.30)*	1.50 (1.23, 1.84)*
Less occurrence of domestic violence	0.89 (0.74, 1.07)	1.09 (0.93, 1.28)	1.36 (1.16, 1.59)*	1.08 (0.90, 1.30)	0.98 (0.84, 1.15)
Use contraceptive method	2.20 (1.88, 2.58)*	1.25 (1.08, 1.45)*	1.96 (1.68, 2.28)*	2.80 (2.40, 3.26)*	2.96 (2.56, 3.42)*
None of the three decisions^2^	1.45 (1.24, 1.70)*	1.42 (1.24, 1.63)*	1.82 (1.58, 2.11)*	1.73 (1.48, 2.03)*	1.92 (1.68, 2.21)*
Paternal education	1.88 (1.57, 2.25)*	2.10 (1.76, 2.51)*	1.62 (1.37, 1.92)*	2.30 (1.94, 2.71)*	1.58 (1.35, 1.85)*
Maternal education	2.02 (1.63, 2.51)*	2.47 (2.00, 3.06)*	1.62 (1.33, 1.98)*	3.29 (2.72, 3.99)*	2.13 (1.77, 2.57)*
Maternal BMI ≥ 18.5	1.37 (1.19, 1.59)*	1.83 (1.59, 2.12)*	1.50 (1.30, 1.73)*	1.74 (1.50, 2.02)*	1.41 (1.23, 1.61)*
Did not have fever in last 2 weeks	1.01 (0.90, 1.15)	1.08 (0.96, 1.21)	1.28 (1.14, 1.44)*	1.38 (1.21, 1.58)*	1.14 (1.01, 1.27)*

^1^Base outcome was No previous birth in the multinomial logistic regression, ^2^Woman’s own health care, making major household purchases, visits to her family or relatives, and *p-value < 0.05. ^†^Logistic regression was used to estimate the odds ratio comparing the Sylhet region with other regions where outcome variables were the indicators given in the first column after adjusting the place of residence, region, and survey time.

## Discussion

With remarkable accomplishments in several health indicators, Bangladesh is yet to achieve the goal of reducing undernutrition among children under the age of 5 years, particularly in the Sylhet region. Through this analysis, we aimed to explore the factors influencing childhood undernutrition and compare the factors between Sylhet, the most vulnerable region, and other regions of Bangladesh.

We found that overall, child reporting fever in recent days, maternal BMI, type of birth, mother’s education, father’s education, household wealth, and geographical region were the common significant associated factors for childhood undernutrition. Having a fever in the 2 weeks leading up to the survey appears to be a significant determinant in childhood undernutrition. This has been established in previous studies in resource-poor settings (18, 19). Infections cause decreased food intake, nutritional losses due to poor digestion, and metabolic disturbances, all leading to undernutrition (19).

Our results show that children of women with no or only primary education are more likely to be affected by any kind of undernutrition. However, we also found a trend in the reduction of low educational qualifications among the surveyed mothers with increased percentages of mothers having secondary or higher secondary education over the decades. Other Bangladeshi studies have reported comparable results, showing that children of mothers with less educational qualification were significantly at greater risk of being undernourished (20, 21). Education enables a mother to receive and process information more effectively (22), empowering a mother to make informed decisions regarding health and nutrition. Also, it might increase their utilization of child health services (23).

Household wealth (the two measures of SES) appeared to be a strong predictor of child malnutrition outcomes. In Bangladesh and other underdeveloped nations, socio-economic indicators are protective factors for child health (12). Children residing in higher-income households are more likely to belong to comparatively food-secure families, have parents with relatively higher education and live in a better area with better access to health facilities (11, 24, 25). All these factors in combination possibly modulate the risk of undernutrition among the children residing in higher wealth index households.

One of our key objectives was to explore if there is any geographical variation in the established common associated factors of undernutrition. We found that the Sylhet division lags in several key risk indicators, namely parental education, maternal BMI, receiving at least four ANC from the medically trained provider, women empowerment, and use of contraceptive methods compared to other regions. UN reports (26), as well as several local studies (11, 13, 27–30), have confirmed the poor performance of the Sylhet division despite having the lowest poverty rate in the country. This has been well documented that along with the highest rates of chronic childhood undernutrition that is stunting, Sylhet also has the lowest female literacy rates, the worst school attendance rates for adolescent girls, the highest gender inequality scores, the worst performance against women’s empowerment indicators, and overall the lowest proportion of empowered women in the nation (31), concurring with our findings. Such a result is very startling as Sylhet is considered a rich region as a vast number of its population lives abroad and sends remittances. However, this is a unique regional characteristic that potentially poses a barrier in several distinctive ways. Geographically, over a fourth of all arable land in Sylhet Division remains uncultivated, and only a single crop is produced on half of the remaining land, making the greater Sylhet region less productive. Furthermore, Haor (wetland) and tea estates are two significantly diverse geographical locations in the Sylhet region where a large portion of the marginalized population resides. It has been reported that about 54% of farmers in greater Sylhet are either marginal landholders or not at all. Affluent non-residents own a major portion of cultivable lands, keeping Sylhet’s farming potential vastly underutilized (32, 33). Related to this, there is a notable temptation among the locals to migrate to a foreign country for a better livelihood. Hence, many poor families lack the willingness to send their children to schools, increasing the high incidence of child labor, paid or unpaid, until they grab any opportunity to go abroad (34, 35).

### Limitations

Our analysis incorporates limitations, similar to other cross-sectional surveys. Due to the study design and cross-sectional data collection in the primary phase, our findings do not allow us to conclude any causal association between the factors and child undernutrition outcomes. Furthermore, the primary data did not include important indicators like child birthweight. We were unable to account for other regional factors such as community-level poverty, physical and financial barriers to health facilities, that may have influenced the associations.

## Conclusion

We conclude that several common indicators play a critical role in regulating different aspects of the nutritional status of the under-five children in Bangladesh. Furthermore, some of the indicators are showing a trend of improvement, but significant regional variation still exists. To achieve a more homogenous improvement across the country, the policymakers must focus on region-specific planning and appropriate intervention.

## Data availability statement

The datasets presented in this study can be found in online repositories. The names of the repository/repositories and accession number(s) can be found below: https://dhsprogram.com/Data/.

## Ethics statement

The studies involving human participants were reviewed and approved by the Bangladesh Demographic and Health Survey received ethical approval from the Inner City Fund (ICF) Macro Institutional Review Board, Maryland, USA and the National Research Ethics Committee of Bangladesh Medical Research Council (BMRC), Dhaka, Bangladesh. Each participant gave informed consent before participating in the survey. The de-identified data for this study were obtained from the DHS online. Institutional ethical approval was not necessary as the study was conducted on anonymous public-use data, which had no identifiable information on the survey respondents. The patients/participants provided their written informed consent to participate in this study.

## Author contributions

KS, MH, and MK: conceptualization. KS and MH: data curation. MH: formal analysis. KS and MK: software and writing – original draft. KS, MK, MH, RR, and TA: writing – review and editing. All authors have read and agreed to the published version of the manuscript.
